# Income, employment, and clinical outcomes in Parkinson’s disease

**DOI:** 10.1016/j.prdoa.2026.100439

**Published:** 2026-04-02

**Authors:** Melissa N. Pacheco, Carmen Uribe, Jaeden Brown, Jacob D. Jones

**Affiliations:** a Department of Psychology, California State University San Bernardino, San Bernardino, CA, USA; b Center on Aging, California State University San Bernardino, San Bernardino, CA, USA

## Abstract

**Introduction:**

Socioeconomic factors such as employment status and household income may influence clinical outcomes in Parkinson’s disease, yet their associations with both motor and non-motor symptoms are not well characterized. This study examined whether employment status and income are associated with cognitive complaints, depressive symptoms, health-related quality of life, and motor severity among individuals with Parkinson’s disease.

**Methods:**

Data were obtained from FoxInsight, and online longitudinal cohort study. Participants aged 40 years and older with Parkinson’s disease who completed self-report questionnaires were included (n = 29,826). Multilevel models examined associations between employment status and household income with cognitive complaints, depressive symptoms, health-related quality of life, and motor severity across up to three annual assessments.

**Results:**

Employment status was significantly associated with all outcomes. Compared with full-time employment, unemployment and retirement were associated with more cognitive complaints, greater depressive symptoms, poorer health-related quality of life, and greater motor severity (all p < 0.05). Part-time employment was generally associated with fewer depressive symptoms and did not significantly differ from full-time employment on several outcomes. Lower household income was significantly associated with greater depressive symptoms, poorer health-related quality of life, and greater motor severity (all p < 0.05), but was not associated with cognitive complaints.

**Conclusion:**

Employment status and household income are important social determinants of both motor and non-motor outcomes in Parkinson’s disease. These findings highlight the potential clinical relevance of socioeconomic factors shaping disease experience and emphasize the need for interventions that address economic and occupational vulnerability.

## Highlights

• Employment status is associated with motor and non-motor outcomes in Parkinson’s disease.
• Lower income predicts worse depression, health-related quality of life, and motor severity in Parkinson’s disease.
• Unemployment and retirement show consistent risk across multiple clinical domains.
• Longitudinal FoxInsight data reveal socioeconomic disparities in Parkinson’s disease outcomes.

## 1 Parkinson’s disease

Parkinson’s disease (PD) is the second most prevalent neurodegenerative disorder worldwide, affecting approximately 200 per 100,000 individuals, with the risk increasing five- to tenfold between the ages of 50 and 90 [1]. Its pathological hallmark includes neural inclusions in the form of Lewy bodies and Lewy neurites, as well as the loss of dopaminergic neurons in the substantia nigra [2]. PD, marked by both motor and non-motor symptoms, includes motor impairments characterized by bradykinesia, a core feature of the disorder, in combination with resting tremor or muscular rigidity, with additional features such as postural instability often emerging later in the disease course [3]. Non-motor symptoms include sleep disturbances such as rapid eye movement sleep behavior disorder, insomnia, and restless legs syndrome [4], as well as neurobehavioral changes (e.g., anxiety, apathy, depression), autonomic dysfunction (e.g., constipation, erectile dysfunction), and sensory impairments (e.g., smell loss, visual impairment) [2], [5]. These symptoms are linked to poorer health-related quality of life (HRQoL), increased cognitive impairment, and greater depression [6].

## 2 Socioeconomic status, aging, and health outcomes

Lower socioeconomic status (SES) has been associated with an increased risk of long-term health issues and accelerated declines in physical, mental, and social functioning, even when controlling for diagnosed health conditions or self-rated health [7]. In fact, approximately 11.3% of individuals aged 60 and older are below the poverty level in the United States [8], which highlights the importance of investigating SES disparities on aging-related impairments, such as depression, cognition, and HRQoL.

## 3 Depression

Xue et al. [9] found that older adults without a stable source of income are at an increased risk of developing depression. They also noted that lower educational attainment, as a component of socioeconomic status, is linked to economic disadvantage, which may contribute to financial stress and increase vulnerability to depression. Additionally, older adults experiencing severe food insecurity–defined as regularly not having enough food or being unable to afford food at times–are more than twice as likely to suffer from depression compared to those without food insecurity [10]. However, Smith et al. [10] found that moderate food insecurity was not found to be associated with depression. Older adults who reported financial difficulties, such as having trouble paying bills or delaying medication due to cost, were significantly more likely to experience high depressive symptoms [11].

## 4 Cognitive health

Research suggests that SES moderates the relationship between age and brain network organization, with lower SES individuals exhibiting reduced brain network differentiation in middle adulthood (35–64 y), which may increase vulnerability to cognitive decline when faced with age-related neurodegeneration [12]. Similarly, a study of adults aged approximately 58–70 found that experiencing four or more years of economic hardship across adulthood was associated with poorer cognitive function, as well as indicators of early aging [13]. Supporting this, a study conducted in Malaysia found that older adults experiencing economic hardship were more likely to exhibit mild to severe cognitive impairment, further suggesting that financial strain may accelerate cognitive decline in later life [14].

## 5 Health-Related Quality of Life (HRQoL)

Hajek and König [15] found that financial hardship in individuals aged 40 and over was associated with increased social isolation and decreased optimism, while overcoming financial hardship improved life satisfaction. Similarly, Dobarrio-Sanz et al. [16] observed that older adults living in poverty often describe life as a constant struggle, with financial hardship affecting nearly every aspect of daily life. Basic needs, such as affording nutritious food and maintaining utilities, become challenging, while financial constraints limit social engagement. Some older adults avoid social interactions due to feelings of embarrassment, further contributing to social isolation and reduced overall HRQoL.

## 6 Income and risk of incident Parkinson’s disease

The association between income and the risk of incident PD has been inconsistent across studies, with studies reporting conflicting and sometimes opposite findings. This variability likely reflects differences in study design, populations, and broader epidemiological heterogeneity [17]. A population-based study found that the annual rate of PD prevalence was significantly different based on income. In urban areas, both the prevalence and incidence of PD were significantly higher among lower-income patients. In rural areas, prevalence of PD was higher for lower-income individuals but incidence was not significantly different [18]. The Unified Parkinson’s Disease Rating Scale (UPDRS) had a significant difference of 6.7 points and the older Americans resource and services disability subscale (OARS) had a significant difference of 5.3 points between high and low socioeconomic status individuals, even when controlling for comorbidity, percentage with PD, and time since diagnosis [19]. Individuals with lower socioeconomic status also had lower rates of PD, with high manual workers having 12% lower rates and low manual workers having 7% lower rates [20]. However, lower socioeconomic status was also associated with higher mortality rates, with hazard ratios indicating significantly decreased mortality for individuals with low- and middle-income levels compared to those in the lowest income group [21].

## 7 Income and clinical outcomes among Parkinson’s disease

There is a paucity of research linking the clinical outcomes of PD, other than motor symptom severity, with patients’ level of income. One study examining deep brain stimulation and Parkinson’s patients, researchers found that patients with higher levels of income had better functional improvement after one year compared to individuals with lower household incomes [22]. Additionally, people with PD who have lower household income experience lower HRQoL [23], however this was in a relatively small (n = 80) and cross-sectional sample of individuals living with PD.

The current paper examines whether employment status and household income, two key indicators of SES, are associated with cognitive complaints, HRQoL, depressive symptoms, and motor severity in individuals with PD. Using longitudinal data from FoxInsight, we investigated these socioeconomic predictors across multiple time points to determine whether differences in economic resources and workforce involvement relate to clinical functioning in PD. By integrating SES factors with core PD outcomes, this study aims to clarify how financial and occupational circumstances may contribute to variability in the lived experience of PD.

## 8 Methods

### 8.1 Participants and procedure

Data for this study was obtained on November 15, 2024 from the FoxInsight database, an online longitudinal cohort study investigating the progression of PD. FoxInsight enrollment began in 2017. For up-to-date information on the study visit https://foxinsight-info.michaeljfox.org/insight/explore/insight.jsp. All participants provided consent prior to study activities.

An initial query resulted in 48,212 participants living with PD. Participants were excluded if they did not complete self-report questionnaires on any occasion (n = 18,065) and if their self-reported age was less than 40 years of age (n = 321). This resulted in a final sample of 29,826 participants. Questionnaires were completed annually for up to 3 years.

### 8.2 Measures

#### 8.2.1 Income and Employment

Employment was self-reported by participants and coded as 1 = employed full-time, 2 = employed part-time, 3 = retired, and 4 = unemployed. Yearly household income was also self-reported by participants and coded as 1 = less than $20,000, 2 = $20,000–$34,999, 3 = $35,000–$49,999, 4 = $50,000–$74,999, 5 = $75,000–$99,999, and 6 = more than $100,000.

#### 8.2.2 Depression

Depressive symptoms were measured using the Geriatric Depression Scale–Short Form (GDS). All participants completed this questionnaire, which asks about the presence or absence of symptoms, such as feelings or sadness or loss of interest. Participants responded with a yes/no to a series of items based on their recent mood and behavior. Higher scores indicate more depressive symptoms.

#### 8.2.3 Quality of life

HRQoL was measured using the Parkinson’s Disease Questionnaire-8 (PDQ-8), an eight-item questionnaire assessing difficulties with activities of daily living, such as dressing or public embarrassment. Participants diagnosed with PD completed this questionnaire at their first study visit and at each subsequent visit. Using a 5-point Likert-type scale, participants rated their difficulty with various tasks, where 0 indicated “no issues” and 4 indicated “always” or “cannot do at all”. Higher scores indicate greater difficulty with HRQoL.

#### 8.2.4 Cognitive complaints

Cognitive complaints were assessed using the 15-item Penn Parkinson’s Daily Activities Questionnaire (PDAQ). This measure evaluated participants’ ability to complete various cognitive tasks, such as multitasking and keeping track of time. Participants responded using a 5-point Likert-type scale, with higher scores indicating better ability to perform cognitive tasks (i.e., fewer complaints).

#### 8.2.5 Motor severity

Motor symptom severity was assessed using Part II of the Movement Disorder Society Unified Parkinson’s Disease Rating Scale (MDS UPDRS-II), a widely used and validated self-reported measure for evaluating motor symptoms in individuals with PD. This section evaluates difficulties with daily activities such as turning in bed, bathing, and dressing. Responses were recorded on a Likert scale, with 0 indicating no issues (normal) and 4 indicating severe impairment.

#### 8.2.6 Statistical analyses

Data were analyzed using multilevel modeling in SPSS, with standardized estimates reported. Four separate models were conducted for each of the following dependent variables: PDAQ, PDQ-8, GDS, and MDS UPDRS-II. Employment status (employed full-time, employed part-time, retired, unemployed) and yearly household income were entered as independent variables. Employed full-time served as the reference group in all models. Additional covariates included sex, age, education, occasion, and motor severity (MDS UPDRS-II). MDS UPDRS-II scores were not included in the model where MDS UPDRS-II was the dependent variable. Motor severity was included as a covariate to account for overall disease burden.

To reduce possible confounds related to the participants’ country of residence, additional sensitivity analyses were conducted where the above analyses were repeated in a subsample of participants (n = 26,420) from the United States only. The four models above were repeated with similar dependent and independent variables.

## 9 Results

Demographic and clinical characteristics are in Table 1. Four separate multilevel models were conducted to examine whether employment status and income were associated with cognitive complaints, HRQoL, depression, and motor severity (Table 2).

**Table 1:** Demographic and clinical characteristics (N = 29,826).

	M	SD	Range
Participant Characteristics			
Age	65.9	9.2	40–90
% Male	43.3%	–	–
% White	97.0%	–	–
% <13 years of education	9.3%	–	–
% 13–16 years of education	54.3%	–	–
% >16 years of education	36.4%	–	–
% Income <$35,000	16.7%	–	–
% Income $35,000-$75,000	30.6%	–	–
% Income >$75,000	52.7%	–	–
% Employed full time	21.1%	–	–
% Employed part-time	7.8%	–	–
% Retired	65.5%	–	–
% Unemployed	5.6%	–	–
% PD Duration < 3 Years	51.3%	–	–
% PD Duration 3–10 Years	35.4%	–	–
% PD Duration > 10 Years	13.1%	–	–
MDS UPDRS-II	11.8	8.1	0–51
PDAQ	49.9	10.4	0–60
GDS	4.4	3.8	0–15
PDQ-8	7.2	5.5	0–32

MDS UPDRS-II = Movement Disorder Society Unified Parkinson’s Disease Rating Scale- Part II; PDAQ = Penn Parkinson’s Daily Activities Questionnaire; GDS = Geriatric Depression Scale- Short Form; PDQ-8 = Parkinson’s Disease Questionnaire-8.

**Table 2:** Associations between income, employment status, and clinical outcomes.

Parameter	PDAQ		PDQ-8		GDS		MDS UPDRS-II	
	B	p	B	p	B	p	B	p
Income	0.026	0.306	−0.072	**<0.001**	−0.127	**<0.001**	−0.190	**<0.001**
Employed Part-Time (vs Full-Time)	−0.046	**0.043**	0.033	0.073	−0.057	**0.019**	0.034	0.203
Retired (vs Full-Time)	−0.170	**<0.001**	0.052	**<0.001**	0.011	0.540	0.371	**<0.001**
Unemployed (vs Full-Time)	−0.315	**<0.001**	0.288	**<0.001**	0.299	**<0.001**	0.618	**<0.001**
Sex	−0.038	**<0.001**	−0.112	**<0.001**	−0.015	0.194	0.249	**<0.001**
Age	0.018	**0.010**	−0.171	**<0.001**	−0.128	**<0.001**	0.035	**<0.001**
Education	0.057	**<0.001**	−0.043	**<0.001**	−0.053	**<0.001**	−0.094	**<0.001**
Motor Severity (MDS UPDRS-II)	−0.526	**<0.001**	0.699	**<0.001**	0.429	**<0.001**	–	–
Occasion	0.004	0.819	−0.002	0.761	−0.002	0.880	0.122	**<0.001**

B = standardized estimates; significant values (p < 0.05) are bolded. PDAQ = Penn Parkinson’s Daily Activities Questionnaire, PDQ-8 = Parkinson’s Disease Questionnaire-8, GDS = Geriatric Depression Scale- Short Form, MDS UPDRS-II = Movement Disorder Society Unified Parkinson’s Disease Rating Scale- Part II.

**Fig. 1:**
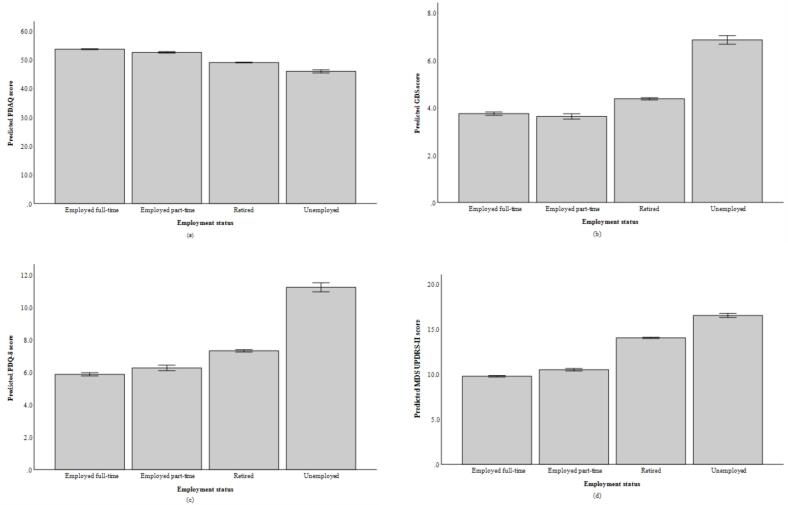
Employment status differences in clinical outcomes among individuals with Parkinson’s disease. Figure depicts model-predicted cognitive complaints, depressive symptoms, health-related quality of life, and motor severity across employment groups. Panels show (a) Penn Parkinson’s Daily Activities Questionnaire (PDAQ), (b) Geriatric Depression Scale-Short Form (GDS), (c) Parkinson’s Disease Questionnaire-8 (PDQ-8), and (d) Movement Disorder Society Unified Parkinson’s Disease Rating Scale- Part II (UPDRS-II) scores.

For cognitive complaints, employment status was a significant predictor. Compared with participants employed full-time, those who were unemployed, retired, or employed part-time reported more cognitive complaints (i.e., lower PDAQ scores) (Fig. 1a). Income was not a significant predictor. Higher education and older age were associated with fewer complaints, while being male was associated with more complaints. Greater motor severity was also associated with more complaints. Occasion was not a significant predictor.

For depressive symptoms, employment status and income were significant predictors. Participants who were unemployed reported more depressive symptoms, while those employed part-time reported fewer symptoms, compared to those employed full-time; retired status was not significant (Fig. 1b). Lower income was also associated with more depressive symptoms. Greater motor severity, lower education, and younger age were each associated with more depressive symptoms, whereas sex was not significant. Occasion was not a significant predictor.

For HRQoL, both employment status and income were significant predictors. Participants who were unemployed or retired reported poorer HRQoL compared with those employed full-time, while part-time employment was not significant (Fig. 1c). Lower income was also associated with poorer HRQoL. Greater motor severity, lower education, younger age, and being male were each associated with poorer HRQoL. Occasion was not a significant predictor.

For motor severity, employment status and income were significant predictors. Participants who were unemployed or retired reported greater motor severity compared with those employed full-time, while part-time employment was not significant (Fig. 1d). Lower income was also associated with greater motor severity. Female sex, lower education, and older age were each associated with greater motor severity. Occasion was also significant, with motor severity decreasing slightly over time.

### 9.1 Sensitivity analyses: United States subsample

Since participants were recruited from various countries with varying levels of economic development, the multilevel models were repeated in a subsample of participants (n = 26,420) from the United States only (Table 3).

**Table 3:** Sensitivity analyses in U.S. participants (n = 26,420).

Parameter	PDAQ		PDQ-8		GDS		MDS UPDRS-II	
	B	p	B	p	B	p	B	p
Income	0.069	**0.007**	−0.095	**<0.001**	−0.142	**<0.001**	−0.193	**<0.001**
Employed Part-Time (vs Full-Time)	−0.039	**0.027**	0.012	0.584	−0.073	**0.008**	0.010	0.736
Retired (vs Full-Time)	−0.167	**<0.001**	0.043	**0.004**	0.012	0.539	0.359	**<0.001**
Unemployed (vs Full-Time)	−0.306	**<0.001**	0.298	**<0.001**	0.338	**<0.001**	0.607	**<0.001**
Sex	−0.037	**0.003**	−0.109	**<0.001**	−0.008	0.517	0.264	**<0.001**
Age	0.017	**0.035**	−0.175	**<0.001**	−0.123	**<0.001**	0.025	**0.008**
Education	0.062	**<0.001**	−0.045	**<0.001**	−0.049	**<0.001**	−0.093	**<0.001**
Motor Severity (MDS UPDRS-II)	−0.521	**<0.001**	0.689	**<0.001**	0.426	**<0.001**	–	–
Occasion	0.010	0.491	0.004	0.622	0.001	0.953	0.115	**<0.001**

B = standardized estimates; significant values (p < 0.05) are bolded. PDAQ = Penn Parkinson’s Daily Activities Questionnaire, PDQ-8 = Parkinson’s Disease Questionnaire-8, GDS = Geriatric Depression Scale- Short Form, MDS UPDRS-II = Movement Disorder Society Unified Parkinson’s Disease Rating Scale- Part II.

For cognitive complaints, findings were largely similar to models with the entire research sample, except that higher income was significantly associated with fewer cognitive complaints. Employment status continued to be a significant predictor. Compared with participants employed full-time, those who were unemployed, retired, or employed part-time reported more cognitive complaints (i.e., lower PDAQ scores).

Regarding depressive symptoms, employment status and income continued to be significant predictors. U.S. participants who were unemployed reported more depressive symptoms, while those employed part-time reported fewer symptoms, compared to those employed full-time; retired status was not significant. Lower income was associated with more depressive symptoms.

In the model with HRQoL, employment status and income remained significant predictors. Participants who were unemployed or retired reported poorer HRQoL compared with those employed full-time, while part-time employment was not significant. Lower income continued to be associated with poorer HRQoL.

Lastly, UPDRS continued to be associated with both employment status and income among the U.S. subsample. Participants who were unemployed or retired reported greater motor severity compared with those employed full-time, while part-time employment was not significant. Lower income was also associated with greater motor severity.

## 10 Discussion

This study investigated the role employment status and income have on cognitive complaints, HRQoL, depression, and motor severity for individuals with PD. Overall, results demonstrated that employment status was significantly associated with outcomes, with unemployment and retirement being linked to worse outcomes compared to being employed full-time; however, these associations may be bidirectional, as worsening clinical symptoms may also contribute to reduced workforce participation. While income did not show up as a significant predictor of cognitive complaints for individuals with PD, it was associated with HRQoL, depression, and motor severity.

### 10.1 Employment and income in relation to cognitive complaints

Employment status was found to be a significant predictor of cognitive complaints, while income was not. PD individuals who were unemployed, retired, or employed part-time reported more cognitive complaints than PD individuals who were employed full-time. This suggests that employment may be associated with better cognitive outcomes in individuals with PD, by serving as a potential protective factor against more severe cognitive complaints. We are not currently aware of any previous studies that have directly examined the relationship between employment status and cognition in PD, however, research in the general aging literature provides helpful context for understanding the role of employment status. For example, Leist et al. [24] found that prolonged periods of time away from work, classified as “unemployment” or “sickness”, predicted poorer cognitive performance in later adulthood versus shorter employment gaps related to training or maternity leave. When considering our findings, this reflects the importance of taking employment status into account when examining potential factors contributing to reported PD cognitive complaints or impairment.

Interestingly, income was not significantly associated with cognitive complaints, which does not align with some prior literature among healthy older adults. For example, prior research has shown that lower socioeconomic status has been linked to reduced brain network differentiation and greater vulnerability to cognitive decline [12], [13]. Our findings also contrast with work from Malaysia, suggesting that financial strain may accelerate cognitive decline and aging [14].

However, these inconsistencies may indicate that income may not fully account for the cognitive benefits that come from continued employment and engagement in cognitively demanding roles. Employment may be associated with cognitive benefits that are not captured by income alone. Employment offers opportunities for routine cognitive engagement, problem solving, and social interaction, which may help buffer cognitive complaints among individuals with PD. This interpretation aligns with aging literature showing that time spent away from cognitively stimulating roles is associated with poorer cognitive outcomes [24].

Aging research supports this perspective, as Finkel et al. [25] found that older adults in more cognitively complex occupations, specifically jobs that require mentally engaging interpersonal interaction, performed better on cognitive tasks prior to retirement. Authors noted that these individuals had higher levels of spatial ability at retirement but then demonstrated a faster decline after retirement, which highlights the impact occupational complexity has during the employed years. This is relevant for PD, because cognitive complaints often involve different cognitive domains that may benefit from the continued cognitive stimulation provided by the environment. Similarly, a recent study utilizing a U.K. sample examined mediator variables involved in the relationship between education level and dementia risk, and reported that occupational complexity explains a much larger portion of the relationship between education level and dementia compared to income [26]. Occupational complexity may play a more direct role in supporting cognitive functioning in the context of PD-related neurodegeneration and this pattern may help partially explain why employment status was found to be a significant predictor of cognitive complaints, compared to income. Taken together, these findings indicate that cognitive demands and social engagement associated with employment may play a more important role in cognitive health than financial income, which may help explain why employment status, but not income, predicted cognitive complaints in our PD sample.

It is also important to consider the potential for reverse causation in this relationship. Individuals with greater cognitive complaints may be less likely to remain in the workforce or may retire earlier due to difficulties managing job-related cognitive demands. As such, the observed association between employment status and cognitive complaints may reflect, in part, the impact of cognitive decline on employment rather than purely protective effect of employment on cognitive functioning. Therefore, these findings should be interpreted as correlational rather than causal.

### 10.2 Employment and income in relation to health-related quality of life

Both employment status and income were significant predictors of HRQoL. Previous studies on SES demonstrated an association between prolonged health issues and lower SES [7]. Studies that specifically discuss PD as a prolonged health issue show that PD is associated with SES factors such as productivity and overall cost. PD patients have worse overall HRQoL, greater productivity loss at work, and higher direct and indirect costs [27]. Taken together, these studies suggest an association between prolonged health issues such as PD and lower SES variables.

While we are not aware of many studies that directly examine HRQoL and SES associations in PD, a previous study shows that higher household income leads to higher QoL in PD. In this study the authors found that QoL was highly related to household income [23]. Additionally, there is a study that shows that lower HRQoL is associated with higher levels of unemployment in PD which is a variable that directly affects SES [28]. Both these findings, and ours, demonstrate that HRQoL and general QoL are related to SES variables.

### 10.3 Employment and income in relation to depression

In our sample, both employment status and income were significant predictors of depression. Unemployment was associated with higher symptoms of depression, which indicates that the complete absence of employment could potentially have a negative effect on mental health outcomes for individuals with PD. Research in PD highlights that many individuals exit the workforce earlier than expected and often experience social withdrawal and loneliness [29], [30]. A more broad and non-PD unemployment study by Paul & Moser [31], also suggests that social contact and social support can help protect against some of the distressing psychological effects of unemployment, which may be important to consider for our PD sample. This research helps clarify why lack of employment may limit social interactions and reduce access to helpful resources, as well as its association with depressive outcomes.

Lower income was also significantly associated with higher symptoms of depression, which greatly aligns with past research. Individuals with PD may be particularly vulnerable to the effects of financial strain, because economic challenges can introduce chronic stress, limit access to treatment, and increase overall daily burden. These factors may contribute to the higher levels of depression observed among lower-income individuals in our sample. For example, previous research has highlighted that older adults without a stable source of income, severe food insecurity, and financial difficulties have an increased risk of developing depression [9], [10], [11].

### 10.4 Socioeconomic disparities in motor symptom severity

In the current study, both employment status and income were significantly associated with motor symptom severity in individuals with PD. Participants who were unemployed or retired reported greater motor severity compared to those employed full-time, and lower household income was also associated with worse motor outcomes. These findings suggest that socioeconomic factors may play an important role in motor functioning among individuals with PD.

Prior PD research supports this pattern. Hemming et al. [19] reported that individuals with higher SES demonstrated significantly better motor outcomes, with differences of 6.7 points on the UPDRS and 5.3 points on the OARS disability subscale compared to lower-SES individuals, even after controlling for comorbidity and disease characteristics. Similarly, recent work examining community-level socioeconomic characteristics exhibited greater motor severity as measured by the UPDRS [32]. Together, these findings indicate that both individual- and community-level socioeconomic factors are associated with motor symptom severity in PD.

Evidence from aging populations further supports the link between socioeconomic disadvantages and physical functioning. In a large longitudinal study, Steptoe and Zaninotto [7] found that lower SES was associated with greater declines in multiple indicators of physical function, including grip strength, gait speed, ability to rise from a chair, and overall physical activity. These findings demonstrate that socioeconomic disparities are linked to physical decline even in the absence of PD, suggesting that individuals with fewer socioeconomic resources may be more vulnerable to motor impairment more broadly. When considered alongside our results, this literature suggests that socioeconomic disadvantage may exacerbate motor symptom severity in PD through mechanisms related to physical functioning and disease management. Of course, it should be acknowledged that there is likely a bidirectional association between socioeconomic resources and motor symptoms severity. As mentioned above, lower SES can lead to worse motor outcomes, but similarly more severe motor symptoms may lead to earlier retirement, loss of wages, greater financial burden (e.g. price of medications and health services) and other adverse SES outcomes [27].

## 11 Limitations and future directions

Several limitations should be noted. First, all measures were self-reported, including income, employment, and clinical outcomes. Although self-report is efficient for large-scale data collection, it may introduce recall bias, reporting error, or subjective interpretation. Second, participants were drawn from an online, volunteer-based cohort, which likely represents a healthier, more affluent, and more technologically literate subset of the PD population. This limits generalizability to more clinically diverse or underserved groups. Additionally, the sample was predominantly White (97%), which further limits the generalizability of these findings to more racially and ethnically diverse populations. Third, our measure of income used broad categorical ranges, which may obscure more subtle financial differences, while employment status did not capture details such as type of work, caregiving responsibilities, or prior occupational history. Finally, the observational design limits our ability to establish causal pathways. This is particularly relevant to the PD population because there may be a bidirectional relationship, whereby socioeconomic disparities may be influenced by disease progression, while disease outcomes may also be shaped by socioeconomic factors. In addition, intervention-focused work is needed to identify strategies that can buffer the negative effects of unemployment or financial strain in PD, such as vocational support, financial counseling, or community-based programs aimed at maintaining HRQoL.

## 12 Conclusion

In sum, this study provides early evidence that both employment status and household income may be important social determinants of clinical outcomes in PD. Unemployment and retirement were consistently linked with poorer outcomes, while lower income was associated with greater depression, worse HRQoL, and more severe motor symptoms. Cognitive complaints appeared to be more closely tied to employment than income, suggesting that engagement in structured roles may help preserve cognitive functioning. These findings highlight the broad impact of socioeconomic disparities in the lived experience of PD and underscore the need for future research and clinical interventions that address economic vulnerability. Efforts to support employment opportunities and financial stability may play an important role in improving well-being and functioning among individuals living with PD.

## CRediT authorship contribution statement

**Melissa N. Pacheco:** Writing – original draft, Visualization. **Carmen Uribe:** Writing – original draft, Visualization. **Jaeden Brown:** Writing – original draft, Visualization. **Jacob D. Jones:** Writing – review & editing, Supervision, Formal analysis, Conceptualization.

## Declaration of competing interest

The authors declare the following financial interests/personal relationships which may be considered as potential competing interests: Jacob D. Jones reports administrative support was provided by Michael J Fox Foundation for Parkinson’s Research. FoxInsight funded the data collection, however this is a secondary analysis of that data. The authors did not receive any compensation for their work. If there are other authors, they declare that they have no known competing financial interests or personal relationships that could have appeared to influence the work reported in this paper.
